# Redefining cancer care: the importance of primary care cancer research in Ireland

**DOI:** 10.1007/s11845-024-03864-6

**Published:** 2025-01-16

**Authors:** Sean O’Regan, Jack Adams, Benjamin M. Jacob, Heather Burns, Patrick Redmond

**Affiliations:** 1 Department of General Practice, RCSI, Dublin, Ireland; 2 HSE National Cancer Control Programme, Dublin, Ireland

**Keywords:** Cancer survivors, Early detection of cancer, Health services accessibility, Ireland, Neoplasms (cancer), Primary health care

## Cancer is a significant public health challenge

Cancer represents a significant public health challenge in Ireland. As of 2022, nearly a third of all deaths were attributed to invasive malignancies [1]. Moreover, one in every two people in Ireland will develop invasive cancer (excluding non-melanoma skin cancer) in their lifetime [2]. Forecasts also indicate a potential doubling of cancer diagnoses between 2010 and 2040, primarily due to an ageing population [3].

Despite these stark statistics, the past three decades have seen Ireland make significant advancements in cancer care, guided by three successive national cancer strategies [1]. These have contributed to reduced mortality rates for the most prevalent cancers and an increase in 5-year age-standardised cancer survival rates from 42 to 65% over a twenty-year period [4]. Despite these achievements, Ireland’s age-standardised incidence and mortality rates remain amongst the highest within the OECD (Organisation for Economic Co-Operation and Development) [5]. This indicates a need for continued innovation in our approach to cancer control.

## Shifting the focus to primary care: prevention and early diagnosis

In recent years, there has been a shift in cancer control strategies towards prioritising prevention and early diagnosis, as the most cost-effective long-term approach to cancer control [6]. The National Cancer Strategy (2017–2026) advocates for robust public education, risk reduction, and early detection programmes, primarily managed within primary care [1]. Simultaneously, there is a global trend towards bolstering primary care as the cornerstone of healthcare due to its accessibility, cost-effectiveness, and preference amongst patients for receiving care closer to home [7]. Reflecting this trend, Ireland’s Sláintecare Programme aims to revolutionise health and social care services by expanding community and primary care infrastructure [8]. Equally, the National Cancer Strategy explicitly calls for an expanded role for general practitioners (GPs) in managing the entire cancer continuum, from prevention and early diagnosis to treatment and ongoing survivor support [1].

## Primary care research and the future

Despite its increasing importance in cancer control, primary care research has historically received less funding compared to laboratory and hospital-based research [9]. From 2016 to 2020, global cancer research funding heavily favoured cancer biology (45%) and drug treatment (20%), with primary care-related themes like diagnosis, screening, monitoring, psychosocial aspects, and survivorship receiving just 15% of total awards [10]. This funding imbalance has contributed to considerable gaps in primary care knowledge and practise.

Addressing these gaps is crucial, and a national research group dedicated to primary care cancer research could be a pivotal step. Such an entity could streamline research efforts, foster meaningful collaborations, and minimise work duplication, thereby supporting researchers to address critical knowledge gaps.

Internationally, this collaborative approach has shown success. Australia’s Primary Care Collaborative Cancer Clinical Trials Group (PC4), The Cancer and Primary Care Research International Network (Ca-PRI), and the UK’s CanTest Collaborative are prime examples of how collaborative efforts can substantially contribute to the field. These groups have successfully bridged the gap between researchers, healthcare practitioners, policymakers, and patients, leading to significant advancements.

In Ireland, a similar momentum is gathering with the formation of a stakeholder group for primary care-focused cancer research [11]. Mobilising existing resources and talent towards a unified research strategy could support enhanced cancer control in Ireland.

### Examples of current research questions

The following are examples of primary care research questions which could address existing knowledge gaps in the Irish context.

### Prevention

Modifiable factors account for 30–50% of cancer incidence [12], and with over 90% of the Irish population visiting their GP annually [13], primary care can significantly impact cancer prevention. The ‘Making Every Contact Count’ (MECC) framework encourages GPs to guide patients towards healthier lifestyles [13]. However, the content, frequency, and effectiveness of these brief interventions in Ireland is unclear. Future research should focus on understanding these interventions, identifying barriers, and exploring ways to enhance the impact of preventative advice.

### Early detection

Early detection of cancer is a key first step towards achieving higher survival rates, reducing treatment severity, and improving quality of life for people living with cancer [14, 15]. Whilst screening enables early detection of some presymptomatic cancer, approximately 85% of cancers are diagnosed after the onset of symptoms [6]. This fact underscores the importance of equipping primary care with robust clinical guidelines and efficient referral pathways. The National Cancer Control Programme’s Rapid Access Clinics (RACs) [16] represent a significant step in this direction. A comprehensive analysis of RAC referrals, including demographic and clinical profiles, is crucial to understand and optimise their utilisation.

### Screening

Screening plays a critical role in reducing cancer incidence and mortality by detecting and treating pre-cancerous conditions and asymptomatic early-stage cancers. However, the uptake of cancer screening in Ireland is inconsistent, influenced significantly by sociodemographic factors [1]. Research into the barriers and attitudes towards cancer screening in Ireland is essential to develop targeted strategies for improving uptake, particularly amongst high-risk groups. Whilst studies pertaining to certain screening programmes, such as the cervical cancer screening programme [17, 18], have been conducted, more comprehensive research across various cancer types is needed.

### Survivorship

The number of cancer survivors has grown by 50% in the past decade with approximately 215,000 cancer patients or former cancer patients alive as of 2021, equivalent to 4.3% of the population [19]. This growth challenges the sustainability of the traditional hospital-centred follow-up model, signalling a shift towards primary care-led survivorship care [6]. Whilst research has been undertaken into the survivorship needs of patients with common cancers, such as prostate cancer, the needs of those with other diagnoses, such as melanoma, testicular, and bladder cancer, have been less comprehensively explored [20]. Further research in these areas is vital to develop evidence-based initiatives addressing the needs of this expanding survivor population.

## Emerging technologies and strategies

The future of primary care cancer research extends beyond the current scope to include emerging technologies that necessitate translational research for clinical application. Notable examples of emerging technologies with implications for primary care include artificial intelligence (AI) models for lung cancer risk stratification using low-dose computed tomography (LDCT) [21], multi-cancer screening through cell-free DNA (cfDNA) tests [22], and personalised medicine approaches as highlighted in Ireland’s National Strategy for Genetic and Genomic Medicine and the Hereditary Cancer Model of Care [23, 24]. These advancements represent a frontier in cancer diagnosis and treatment, requiring a focused strategy to translate them from research into clinical practise effectively.

## Conclusion

As the landscape of cancer care evolves both in Ireland and globally, it presents both challenges and opportunities. The anticipated rise in cancer incidence demands proactive measures to strengthen the Irish healthcare system. By recognising the critical role of primary care across the cancer care continuum, and adopting a strategic approach to investment in research, Ireland can develop and implement evidence-based policies and strategies. This approach will not only mitigate the impacts of increasing cancer incidence but also position Ireland at the forefront of innovative and effective cancer care.
